# Virulence Genes and Antimicrobial Resistance Profiles in *Aeromonas hydrophila* and *Aeromonas dhakensis* Isolated from the Brazilian Food Chain

**DOI:** 10.3390/microorganisms13081851

**Published:** 2025-08-08

**Authors:** Emily Moraes Roges, Veronica Dias Gonçalves, Marcelle da Silva Rodrigues, Marcia Lima Festivo, Paulo Henrique Ott, André Luiz Araujo, Salvatore Siciliano, Lucia Helena Berto, Maria Helena Cosendey de Aquino, Dalia dos Prazeres Rodrigues

**Affiliations:** 1 Laboratório de Referência Nacional em Enteroinfecções Bacterianas/Laboratório de Enterobactérias, Instituto Oswaldo Cruz, Fundação Oswaldo Cruz (FIOCRUZ), Rio de Janeiro 21040361, RJ, Brazil; 2 Programa de Pós-graduação em Higiene Veterinária e Processamento de Produtos de Origem Animal, Faculdade de Medicina Veterinária, Universidade Federal Fluminense (UFF), Niterói 24230340, RJ, Brazil; 3 Laboratório de Biodiversidade e Conservação (LABeC), Universidade Estadual do Rio Grande do Sul (UERGS), Unidade Litoral Norte, Osório 95520000, RS, Brazil; 4 Fundação Instituto de Pesca do Estado do Rio de Janeiro (FIPERJ), Escritório Regional da Costa Verde, Angra dos Reis 23900560, RJ, Brazil; 5 Departamento de Ciências Biológicas, da Escola Nacional de Saúde Pública, Fundação Oswaldo Cruz (FIOCRUZ), Rio de Janeiro 21041210, RJ, Brazil; 6 Coordenação Geral de Laboratórios de Saúde Pública (CGLAB), Ministério da Saúde, Brasília 70719040, DF, Brazil

**Keywords:** Aeromonads, *A. hydrophila*, *A. dhakensis*, virulence, antimicrobial resistance, public health

## Abstract

*Aeromonas hydrophila* and *A. dhakensis* are ubiquitous microorganisms, widespread in aquatic environments, and can cause severe infections in humans and animals. This study aimed to determine the diversity of virulence genes *aer*A, *hly*A, *act*, and *alt* through polymerase chain reaction and the antimicrobial resistance through disk diffusion test of 101 *A. hydrophila* and 34 *A. dhakensis* strains from environmental, animal, and human sources gathered between 2016 and 2019 at the National Reference Laboratory for Enteric Diseases. Overall, the virulence gene distribution was *act* in 35.5% of the samples, *alt* in 40.7%, *aer*A in 42.2%, and *hly*A in 44.5%. Our results revealed that 76.3% of the 135 isolated *Aeromonas* exhibited at least one of the genes above. 76.3% of *A. hydrophila* and 76.5% of *A. dhakensis* exhibited virulence genes distributed among 15 and 12 virulence profiles, respectively. Antimicrobial resistance was observed in 86% of the strains (87.1% in *A. hydrophila* and 82.4% in *A. dhakensis*), with higher rates of resistance to Nalidixic acid (69.3%), Imipenem (31.1%), and Sulfamethoxazole-trimethoprim (15.5%). The occurrence of virulence genes and antimicrobial resistance in *A. hydrophila* and *A. dhakensis* from different sources indicates their diversity and pathogenicity, reinforcing that they can be a potential health risk source.

## 1. Introduction

*Aeromonas* spp. are emerging pathogens capable of colonizing and infecting several hosts. Widespread in various habitats, *Aeromonas* spp. are mainly present in aquatic environments but also in soil, food, and animals [1,2]. These bacteria can be pathogenic to fish and other animals and are also involved in multiple infections in immuno-competent and immunocompromised humans [3].

The genus *Aeromonas* belongs to the family Aeromonadaceae, and currently, more than 30 species and seven subspecies are recognized within it [4]. Aeromonads are facultatively anaerobic, Gram-negative, rod-shaped, and non-spore-forming bacteria of approximately 1–3 μm, oxidase-positive, glucose-fermenting, and capable of tolerating concentrations of NaCl from 0.3% to 5% [1]. Gonçalves-Pessoa et al. [2] reported that *Aeromonas* represents the modern challenges in clinical microbiology: constant change in virulence triggered by the acquisition of genetic determinants through lateral transference and the emergence of multidrug-resistant (MDR) strains.

*Aeromonas* spp. can cause several diseases in humans and animals. Their genetic plasticity and diversity of virulence factors make Aeromonads very versatile microorganisms [2]. Since virulence is complex, its distinctive virulence factors contribute to the infection process as the efficacy of the host immune system decreases [5]. *Aeromonas* species produce a heterogeneous range of virulence factors, including hemolysins, enterotoxins, cytotoxins, and adhesives, which have been implicated in the pathogenicity of these bacteria [5,6,7].

Numerous cases reported in the literature confirm that Aeromonads are notable pathogens in human and animal infections [4,6,8,9,10,11,12,13]. These bacteria also contribute to economic losses in aquaculture due to their ability to infect fish and other aquatic organisms [14]. Their pathogenicity is complex and involves various virulence factors, including extracellular enzymes, siderophores, enterotoxins, hemolysins, pili, flagella, and membrane proteins, which allow them to adapt to different environments and hosts [15]. This genetic plasticity and the ability to produce multiple virulence factors contribute to their versatility and success as pathogens, especially as host immune defenses become compromised [2,5,6,7].

Among the specific virulence mechanisms, *Aeromonas* species produce a range of toxins, such as cytotonic enterotoxin (*act*), heat-labile cytotonic enterotoxin (*alt*), hemolysin (*hly*A), and aerolysin (*aer*A), all of which play distinct roles in pathogenesis [3,16]. Additionally, these bacteria secrete various extracellular enzymes like lipase, protease, DNase, and hemolysin, which help degrade host tissues and evade immune responses. These virulence factors contribute to tissue damage, immune evasion, and the establishment of infection by interacting with and disrupting host cellular processes [1,5].

*Aeromonas* are described as having an intrinsic resistance to ampicillin, amoxicillin-clavulanate, and cefazolin [3,17]. Generally, most *Aeromonas* strains present in vitro susceptibility to aminoglycosides, third and fourth-generation cephalosporins, carbapenems, quinolones, and sulfamethoxazole-trimethoprim, although recent studies [6,8,16,17,18,19,20] described cases of resistance to these antimicrobial drugs. The role of members of this genus in the dissemination of antibiotic resistance genes is related to the recognized genomic plasticity and ecology, with habitats including water sources associated with human activities, and *Aeromonas* spp. has been studied as an indicator of the dissemination of antimicrobial resistance (AMR) in water and fish [21,22,23].

*Aeromonas hydrophila*, *Aeromonas caviae*, *Aeromonas veronii* biovar sobria, and *Aeromonas dhakensis* are frequently reported as disease-causing primary or opportunistic pathogens in humans and animals [3,24] has been regarded as important *Aeromonas* species which are implicated in a variety of human diseases [25]. However, according to Chen et al. [26], the importance attributed to *A. hydrophila* should be re-evaluated due to the changing taxonomy. 

A reclassification of the genus *Aeromonas* taxonomy was proposed in 2013, recognizing the newly described species *Aeromonas dhakensis*, previously known as *Aeromonas hydrophila* subsp. *dhakensis*, then misclassified as *Aeromonas aquariorum* [27]. *Aeromonas dhakensis* is an emerging pathogen worldwide and can cause invasive human diseases. Chen et al. [28] indicated that *Aeromonas dhakensis* is widely distributed in the environment and can cause a variety of infections both in humans and animals, especially in coastal areas.

Between 2016 and 2019, the National Reference Laboratory for Enteric Diseases (IOC/FIOCRUZ/Brazil) received strains of *Aeromonas hydrophila* and *A. dhakensis* isolated from several sources. Those strains were evaluated to gain insight into their virulence and antimicrobial resistance. This study investigates the clinical, veterinary, and environmental strains of *A. hydrophila* and *A. dhakensis* and the association of four putative virulence genes from hemolysins and enterotoxins and antimicrobial resistance with source and species.

## 2. Materials and Methods

### 2.1. Strain Selection

A total of 135 *A. hydrophila* (n = 101) and *A. dhakensis* (n = 34) strains isolated from environmental (n = 37) (sewage water), animal (n = 81) (seabirds, marine mammals, fishes, and scallops), and human clinical sources (n = 17) (Table 1) from 2016 to 2019 were analyzed at National Reference Laboratory for Enteric Diseases—NRLED, Oswaldo Cruz Institute, FIOCRUZ. Environmental sewage samples were collected during the monitoring of enteropathogens in water. Fish and scallops were from their natural habitat, and the seabird and marine mammal strains were obtained through monitoring programs at FIOCRUZ. The human samples were isolated from patients with clinical symptoms and sent to NRLED by Public Health Laboratories.

### 2.2. Genus Aeromonas Identification Using the GCAT-PCR (237 bp) and Biochemical Confirmation of the Species

Following the procedures described by Janda and Abbott [3], Martin-Carnahan, and Joseph [29] for enrichment, isolation, and identification, the samples were enriched in Alkaline Peptone Water (APW) containing 1% sodium chloride (NaCl), then were sown in Glutamate Starch Phenol-Red Agar medium (Merk, Darmstadt, Germany).

Yellow colonies (*Aeromonas*-like) were submitted to the oxidase test. The oxidase-positive colonies were screened into Kligler Iron Agar (Difco, Detroit, MI, USA) and Lysine Iron Agar (Difco) and identified to the species level by several biochemical tests: resistance to O/129 vibriostatic agent (2, 4 diamino-6, 7 diisoprylpteridine) in 10 µg and 150 µg concentrations. Growth in 0–3% (*w*/*v*) NaCl, glucose gas production, acid production from the fermentation of carbohydrates), Voges–Proskauer test, esculin hydrolysis, lysine and ornithine decarboxylase, and arginine dehydrolase.

Glycerophospholipid-Cholesterol Acyltransferase (*gcat*) gene was amplified using primer pair as reported previously [12,23]. The presence of this gene (237 bp) was visualized on 2% agarose gel (Sigma, St Louis, MO, USA) stained with Ethidium Bromide [30].

### 2.3. Determination of Antimicrobial Susceptibility

Antimicrobial susceptibility testing was performed using the disk diffusion method according to the Clinical and Laboratory Standard Institute (CLSI) recommendations for *Aeromonas* species [17] and *Enterobacteriaceae* [31]. The antimicrobials drugs, Nalidixic Acid (NAL) 30 µg; Ceftazidime (CAZ) 30 µg; Cefoxitin (FOX) 30 µg; Ceftriaxone (CTX) 30 µg; Ciprofloxacin (CIP) 5 µg; Chloramphenicol (CHL) 30 µg; Gentamicin (GEN) 30 µg; Imipenem (IPM) 10 µg; Meropenem (MEM); Nitrofurantoin (NIT) 300 µg; Sulfamethoxazole-trimethoprim (SXT) 1.25/23.75 µg; and Tetracycline (TCY) 30 µg. *Escherichia coli* ATCC 25922 was used for quality control of the antimicrobial susceptibility test.

### 2.4. Polymerase Chain Reaction (PCR) of Virulence Genes

DNA extraction was performed using a commercial kit (DNA Dnaeasy Tissue—Qiagen, Hilden, Germany) following the manufacturer’s instructions. The DNA amplification step was conducted to investigate the virulence genes hemolisyn (*hly*A—597 bp) [32], cytotoxic enterotoxin (*act*—232 bp), heat-labile cytotonic enterotoxin (*alt*—442 bp) [33], and aerolysin (*aer*A—431 bp) [34]. Eight microliters of PCR product mixed with 5×gel loading dye was loaded onto an agar gel in Tris-Borate-EDTA buffer (0.89 M Tris, 0.89 M Boric acid, 0.02 M EDTA, pH 8.0), and a 100 bp DNA ladder (Invitrogen by Thermo Fischer Scientific, Carlsbad, CA, USA) was used as a molecular weight marker. To view the PCR products, 8 µL of each amplicon was used for electrophoresis in agarose gel 2% (Sigma) and visualized by a UV transilluminator (ImageQuant, GE, Chicago, IL, USA)).

## 3. Results

GCAT-PCR-positive tests confirmed all isolates as genus *Aeromonas*. The biochemical tests confirmed the previous phenotypic identification of species *A. dhakensis* and *A. hydrophila*.

Of the 135 isolates, only 32 (8 *A. dhakensis* and 24 *A. hydrophila*) did not express any of the four virulence genes evaluated. The enterotoxin gene *act* was detected in 35.5% (14 *A. dhakensis* and 34 *A. hydrophila*) of the isolates (48/135). The enterotoxin gene *alt* was detected in 40.7% (11 *A. dhakensis* and 44 *A. hydrophila*) of the isolates (55/135). The aerolysin gene *aer*A was detected in 42.2% (16 *A. dhakensis* and 41 *A. hydrophila*) of the isolates (57/135). The hemolysin gene *hly*A was detected in 44.5% (17 *A. dhakensis* and 43 *A. hydrophila*) of isolates (60/135), as presented in Table 2.

In our human-origin Aeromonads, the gene *hly*A was most frequent, found in 65% (11/17) of isolates. The genes *act* and *hlyA* were more frequent among environmental strains, resembling 27% (10/37) positivity for both genes. Finally, among the animal-origin strains, the genes *alt* and *aer*A were more frequent, with 53% (43/81) and 49.4% (40/81) positivity for each gene, respectively.

The analysis of virulence profiles based on the distribution of the four virulence-associated genes (*act*, *alt*, *aer*A and *hly*A) among the environmental, human, and animal *Aeromonas* species revealed 15 virulence profiles and (Table 3), 16 isolates (one environmental and 11 animal *A. hydrophila* and four animal *A. dhakensis*) amplified all four genes. The profile *act*-*alt*-*aer*A*-hly*A was the most prevalent among 16 isolates. The second most prevalent profile was *aer*A*-hly*A found among nine *A. hydrophila* and three *A. dhakensis* isolates.

Of the 135 *Aeromonas* isolates, 86% (116/135) showed resistance to at least one of the antimicrobial drugs studied. The highest percentage of antimicrobial resistance was to Nalidixic Acid, the second highest was to Imipenem, and Cefoxitin was the third. The results of the 135 *Aeromonas* isolates against the 11 antimicrobial drugs are shown in Table 4. Higher rates of resistance to Nalidixic Acid and Cefoxitin were observed in *A. dhakensis*, 64.2% and 35.3%, respectively. Otherwise, a higher resistance to Imipenem was found in *A. hydrophila*.

Table 4 shows the percentages of AMR in *A. hydrophila* and *A. dhakensis* by antimicrobial drug, and we can see that sensitivity to Cefotaxime, Ciprofloxacin, Gentamycin, and Chloramphenicol was higher than 90% among the 135 isolates. The absence of strains resistant to Cefotaxime and Ciprofloxacin among the *A. dhakensis* isolates is particularly noteworthy.

Antimicrobial resistance to second-generation cephalosporin—Cefoxitin—was higher than that found in third-generation cephalosporins—Ceftazidime and Cefotaxime—in both *A. dhakensis* and *A. hydrophila*. Of the 135 strains, 24 strains (17.7%) exhibited MDR phenotype (Table 5)—antimicrobial resistance to at least one antimicrobial drug in three or more antimicrobial categories; 20% (7/34) of *A. dhakensis* strains and 16.8% (17/101) of *A. hydrophila*.

## 4. Discussion

Based on the results, it appears that 76.3% of the isolates carry virulence genes that are associated with pathogenicity. When evaluating each species separately, 76.5% of *A. dhakensis* (26 strains out of 34) and 76.3% of *A. hydrophila* (77 strains out of 101) tested positive for at least one of the virulence genes investigated. The results indicated that most tested strains, whether *A. dhakensis* or *A. hydrophila*, can potentially lead to illness.

The high prevalence of enterotoxin genes (*act* and *alt*) suggests that these isolates may be capable of causing foodborne illness, as some studies have previously implicated these toxins in outbreaks of gastrointestinal disease [1,4]. The amplification of the aerolysin gene (*aer*A) and the hemolysin gene (*hly*A) also suggests that these isolates may be capable of causing tissue damage and other symptoms associated with *Aeromonas* infections.

Many *Aeromonas* strains bear virulence genes linked to their pathogenicity, such as enterotoxins, hemolysins, and aerolysins [35]. Houssain et al. [36] found that 52 *Aeromonas* spp. isolated from ornamental guppy carried at least two of twelve virulence genes evaluated, with *the act* (84.6%), *hly* (80.8%), and *aer* (73.1%) as the most frequent. Similarly, a study conducted in Taiwan [10] found that all their *Aeromonas* strains carried at least one virulence gene, and more than half harbored *act*, *hlyA*, and *fla*.

Some studies have reported similar findings to our results. For example, a study conducted in China [37] reported a high prevalence of the *hly*A gene (65.5%) in bacteremia caused by aeromonads. Soltan Dallal et al. [38] reported the *aer*A gene in 83.3% of *Aeromonas* strains isolated from children with diarrhea in Tehran, Iran. Still, it is worth noting that the prevalence of virulence genes in *Aeromonas* strains can vary depending on the geographic location, source of isolation, and detection method [35].

Overall, the prevalence of virulence genes in *Aeromonas* isolates appears to be high across different regions worldwide, which implies that these bacteria can potentially cause a range of infections in humans and animals [39]. A study comparing Aeromonads from Singapore and Malaysia [40] revealed *A. hydrophila* and *A. dhakensis* as more virulent than other species, displaying different combinations of nine virulence genes. Another study from Nigeria reported an intermediate prevalence of *hly*A in *Aeromonas* isolates from abattoir and aquaculture environments (respectively 43.8% and 38.5%) [41]. A Brazilian study with ready-to-eat foods found that 41.7% of the *Aeromonas* isolates had *act* gene, and 38.9% had *alt* and *hly*A [42]. Another study from Brazil reported a higher prevalence of *alt* (81%) in *Aeromonas* from clinical and environmental samples of an outbreak [43].

The results suggest that the prevalence of virulence genes in *Aeromonas* isolates varies depending on the source of the sample. The gene *hly*A was the most prevalent among isolates of human origin, indicating that this gene may play a significant role in the virulence of *Aeromonas* strains that infect humans. Similarly, previous studies show that the hemolysin produced by the *hly*A gene can cause damage to human cells and tissues [2,3,6].

However, among the environmental strains, the *act* and *hly*A genes showed a higher percentage of isolation, suggesting that these genes may be meaningful for the survival and adaptation of *Aeromonas* strains under environmental conditions. This aligns with previous studies suggesting that these genes may play a role in the metabolism and adaptation to different environmental conditions of *Aeromonas* strains [1,2,44].

Among the isolates of animal origin, there was a higher prevalence of *alt* and *aer*A genes. These genes have been associated with the production of enterotoxins and pore-forming toxins, which can cause diarrhea and other gastrointestinal symptoms in animals. This finding suggests that these virulence genes could be particularly significant in the pathogenesis of *Aeromonas* infections in animals [1,45,46].

Chen et al. [26] identified the same frequency of *aer*A in bacteremic isolates of *A. dhakensis* and *A. hydrophila.* Notably, the mortality rate of the patients with *A. dhakensis* bacteremia was higher compared to those with bacteremia due to other *Aeromonas* species at Taiwan’s Medical Center.

Following the investigation of *Aeromonas* isolated from intestinal and extra-intestinal infections at a general hospital in Beijing, the pattern of virulence genes varied among *Aeromonas* species. In a comparison between *A. dhakensis* and *A. hydrophila*, the genes *act*, *alt*, *hlyA*, and *aerA* were more frequently found in *A. dhakensis* [4], which contrasts with our results from human-origin Aeromonads.

Li et al. [47] analyzed 257 strains of *Aeromonas* from diarrheic patients and environmental samples in China. They found that the *act* gene was present in 64.5% of the samples, while the *hly*A, *alt*, and *aer*A genes were detected in 33.1%, 31.5%, and 10.5% of *Aeromonas* strains, respectively. These findings align with the results of Zhou et al. [4], who reported frequencies of 42.7% for *hly*A and 19.8% for *aer*A genes. However, our study shows that *hly*A and *aer*A have the highest frequencies, while *act* is the least prevalent in human samples.

Understanding the virulence potential of Aeromonads is crucial, and an effective tool for this is analyzing gene profiles that consider distributing virulence-associated genes among environmental, human, and animal species. We identified 15 virulence profiles by analyzing the presence or absence of four virulence-associated genes: *act*, *alt*, *aer*A, and *hly*A.

The most common profile, which included all four virulence genes (*act-alt-aerA-hlyA*), was found in 16 strains of *A. hydrophila* (1 environmental and 15 animal) and 4 strains of *A. dhakensis* (all of animal origin). The second most frequent profile, *aerA-hlyA,* occurred in nine *A. hydrophila* strains and three *A. dhakensis* strains.

The analysis of virulence profiles presented in Table 3 identified some interesting points. First, while 16 isolates exhibited all four virulence genes, none were derived from human origin. Second, the human-origin strains had a lower percentage of negative results in the search for virulence genes. Third, our data show that *A. hydrophila* and *A. dhakensis* isolated from different sources can share virulence properties. Fourth, our *A. dhakensis* strains are less virulent than our *A. hydrophila*, in divergence from some comparative studies in the literature [26,28,37].

This pattern may suggest that environmental and animal isolates face selective pressures that favor the retention or expression of a broader set of virulence genes, potentially to survive in diverse hosts or harsher environmental conditions. In contrast, human-pathogenic strains may rely on a more specialized set of virulence factors specifically adapted to human tissue or immune defenses, possibly making the full complement of these genes less necessary for pathogenesis in human hosts [15,48].

Some elements can contribute to the distinct virulence profiles observed in human infections compared to those in aquatic animals and environmental samples. Since *Aeromonas* spp. are opportunistic pathogens, adaptable to different conditions, variations in ecological niches can lead to variations in selective pressures, resulting in the acquisition or loss of specific virulence genes. Aeromonads are recognized for their genomic flexibility, harboring a wide repertoire of virulence factors that can vary significantly among strains. These bacteria are known to engage in horizontal gene transfer, which can lead to significant differences in virulence gene content among strains isolated from different sources. Also, human-associated *Aeromonas* strains may have developed specific traits that enhance their ability to infect and cause disease in humans, which may not be necessary for strains that primarily inhabit non-human hosts [48,49,50]. These factors may contribute to the variations in virulence patterns between human and non-human *Aeromonas* strains. Further comparative studies on gene expression under different environmental stresses could clarify this hypothesis and help determine how virulence gene profiles vary according to host and ecological pressures.

Several studies have examined the distribution of virulence-associated genes in *Aeromonas* species, and genes *hly*A, *aerA*, *act*, and *alt* belong to the most studied virulence genes in the literature [4,8,9,35,39,48,51]. The distribution of these virulence-associated genes in *Aeromonas* can vary significantly based on the source and geographic location of the isolates.

Our study identified the *act-alt-aerA-hlyA* and *aerA-hlyA* virulence profiles as prevalent. However, it is essential to recognize that the prevalence of virulence genes and profiles can differ considerably across study populations and geographic regions. Oliveira et al. [51] reported 15 virulence gene combinations in aeromonads from ornamental fish. Their *Aeromonas* strains were more than 50% positive for the studied genes, including *act*, *aer*A, *alt*, and *hly*A, and *hlyA+/aer+/act+/alt+* was the main virulence profile in *A. hydrophila* (63.7%). Additionally, a study about the diversity and dynamics of *Aeromonas* spp. in Bangladesh coastal waters [52] identified 15 virulence profiles, of which 10 were associated with non-enterotoxigenic strains, as further confirmed by animal experiments.

*Aeromonas* can act as a reservoir of genes encoding resistance to antimicrobial drugs, as they are widespread in aquaculture production environments and contribute to the dissemination of antimicrobial resistance determinants, such as carbapenemases and resistance plasmids [35,53]. The high percentage of antimicrobial resistance among the 135 *Aeromonas* isolates in this study is concerning, as it suggests that these bacteria are becoming increasingly resistant to commonly used antimicrobials. The discovery of MDR strains in samples from wild animals and sewage suggests *Aeromonas* may play a role in spreading antibiotic resistance in the environment, seafood, and human communities.

Zhou et al. [4] reported that many of their samples showed high resistance to cefoxitin, imipenem, and nalidixic acid. We also found similar results, with the highest resistance rates for these drugs. However, unlike our results, where only 20% of *A. dhakensis* and 16.8% of *A. hydrophila* isolates were MDR, Zhou et al. [4] identified more than 80% of MDR strains between *A. hydrophila* and *A. dhakensis*.

Teodoro et al. [42] reported that cefepime, ceftazidime, and ciprofloxacin were highly effective, with all tested isolates showing susceptibility. In contrast, our findings show a partial disagreement, as 15% of *Aeromonas* spp. were resistant to ceftazidime and 2% to ciprofloxacin, and cefepime was not evaluated in our study.

The susceptibility results for cefotaxime, ciprofloxacin, gentamicin, and chloramphenicol are encouraging, as these are important antibiotics used for treating various infections. The susceptibility of all 34 *A. dhakensis* isolates to cefotaxime and ciprofloxacin is notable, as these antibiotics are commonly used to treat bacterial infections in humans [11,54]. However, the higher resistance to cefoxitin, compared to third-generation cephalosporins like ceftazidime and cefotaxime in both *A. dhakensis* and *A. hydrophila*, is a concern. High resistance rates to cefoxitin in *Aeromonas* species may indicate the presence of genes conferring resistance to multiple classes of beta-lactams, which could limit treatment options for infections caused by these bacteria and contribute to the spread of these genes to other microorganisms [55,56].

The finding that 17.7% of strains are MDR is also concerning, as it indicates these bacteria can evade multiple classes of antibiotics. The higher proportion of MDR strains among *A. dhakensis* compared to *A. hydrophila* is notable and underscores the need for continued surveillance of antimicrobial resistance patterns in these bacteria.

Some authors propose that *A. dhakensis* should be the focus of future research, as it may harbor higher numbers of virulence genes, exhibit higher drug resistance, and are involved in intestinal and extra-intestinal infections [4,15,28]. Although our results show a higher percentage of *A. hydrophila* MDR strains compared to *A. dhakensis*, which is consistent with other studies suggesting that *A. hydrophila* is more likely to develop antimicrobial resistance than other *Aeromonas* species [57,58,59,60,61], this emphasizes the need for continued surveillance to ensure effective treatment options and to prevent the spread of MDR strains.

Moreover, the results of this study emphasize the relevance of tracking both virulence genes and antimicrobial resistance in *Aeromonas* spp. across various species and isolation sources. Our findings show patterns that warrant further exploration, particularly regarding how these virulence factors contribute to pathogenicity and evolve across different environments and host types. Future research prioritizing a deeper analysis of the genetic virulence and resistance mechanisms can contribute more effectively to preventing and controlling infections caused by *Aeromonas* spp. and preserving the effectiveness of available antimicrobial treatments.

## 5. Conclusions

Overall, these results emphasize the importance of considering the sample source when studying the prevalence and distribution of virulence genes in *Aeromonas* isolates. The differences observed in the prevalence of specific genes among the different sources of isolates may reflect differences in the pathogenesis, adaptation, and host range of *Aeromonas* strains. It is suggestive that the prevalence of virulence genes among *Aeromonas* isolates can vary depending on the source of isolation, with different sets of virulence genes more commonly found in isolates of human, environmental, and animal origin. It is important to note, however, that the mere presence of virulence genes does not necessarily mean that these strains are pathogenic, as many factors can influence the expression of these genes. Nonetheless, identifying different virulence profiles can provide information about the pathogenic potential of different *Aeromonas* strains, provide valuable insights into Aeromonads’ virulence, and inform further research on the mechanisms of pathogenicity of these bacteria. The high susceptibility of most of the *Aeromonas* isolates to Cefotaxime, Ciprofloxacin, Gentamycin, and Chloramphenicol is promising and suggests that these antimicrobials could effectively treat infections caused by these bacteria. The presence of MDR strains is also worrying, as it indicates that these bacteria have acquired resistance to several antimicrobial drugs, which can make it more difficult to treat infections caused by these strains. This highlights the need for continued surveillance and monitoring of antimicrobial resistance among *Aeromonas* species to ensure that effective treatment options are available and to prevent the spread of MDR strains.

## Figures and Tables

**Table 1:** *A. hydrophila* and *A. dhakensis* strains distributed by source.

Source		*A. dhakensis*	*A. hydrophila*
Human (n. 17)	Blood	1	0
	Fecal swab	3	1
	Feces	3	4
	Wound exudate	0	1
	Leg abscess	0	1
	Lung cavity secretion	0	1
	Peritoneal fluid	0	1
	Respiratory wound	0	1
Environment (n. 37)	Sewage water	13	24
Animal (n. 81)	Fishes and Scallop		
	*Cichla ocellarus (Peacock bass)*	0	2
	*Isopisthus parvipinnis* (Bigtooth corvina)	0	2
	*Micropogonias furnieri* (Croaker)	0	2
	* Mugil liza * (Lebranche mullet)	8	32
	* Rachycentron canadum * (Cobia)	0	2
	*Rhizoprionodon porosus* (Caribbean sharpnose shark)	0	4
	* Nodipecten nodosus * (Lion’s paw scallop)	1	3
	Seabirds		
	*Croicocephalus maculipennis* (Brown-hooded gull)	0	1
	*Fregata magnificens* (Magnificent frigatebird)	0	1
	*Larus dominicanus* (Kelp gull)	0	1
	*Phalacrocorax brasilianus* (Neotropic cormorant)	2	12
	*Sula leucogaster* (Brown booby)	0	2
	Marine Mammals		
	*Balaenoptera* sp. (Whale)	0	1
	*Inia araguaensis* (Araguaian river dolphin)	0	1
	*Physeter macrocephalus* (Sperm whale)	0	1
	*Trichechus inunguis* (Amazonian manatee)	3	0

**Table 2:** Distribution of virulence genes in *A. dhakensis* and *A. hydrophila* by source.

Species	Source—n°	Positive Strains—n° (%)			
		*act*	*alt*	*aer*A	*hlyA*
*Aeromonas dhakensis* (n = 34)	7; human	1 (14.3)	1 (14.3)	3 (42.8)	4 (57.1)
	14; animal	9 (64.3)	7 (50)	10 (71.4)	9 (64.3)
	13; sewage water	4 (30.8)	3 (23)	3 (23)	4 (30.8)
*Aeromonas hydrophila* (n = 101)	10; human	2 (20)	4 (40)	6 (60)	7 (70)
	67; animal	26 (38.8)	34 (50.7)	30 (44.8)	30 (44.8)
	24; sewage water	6 (25)	6 (25)	5 (20.8)	6 (25)

*Act*—cytotoxic enterotoxin, *alt*—heat-labile cytotonic enterotoxin; *aer*A—aerolysin; *hly*A—hemolysin; n°—number.

**Table 3:** Virulence profiles in *Aeromonas* spp. according to source and species.

	*Aeromonas dhakensis*	*Aeromonas hydrophila*	Total
Sewage water strains	n = 13	n = 24	n = 37
*act*	1 (7.7%)	2 (8.3%)	3 (8.1%)
*act*- *aer*A	1 (7.7%)	1 (4.2%)	2 (5.4%)
*act*- *aer*A*-hly*A	1 (7.7%)	0	1 (2.7%)
*act*-*alt*-*aer*A*-hly*A	0	1 (4.2%)	1 (2.7%)
*act*-*alt*-*hly*A	1 (7.7%)	1 (4.2%)	2 (5.4%)
*act*-*hly*A	0	1 (4.2%)	1 (2.7%)
*aer*A	0	3 (12.5%)	3 (8.1%)
*alt*	1 (7.7%)	2 (8.3%)	3 (8.1%)
*alt*-*aer*A*-hly*A	1 (7.7%)	0	1 (2.7%)
*alt*-*hly*A	0	2 (8.3%)	2 (5.4%)
*hly*A	1 (7.7%)	1 (4.2%)	2 (5.4%)
Negative	6 (46.1%)	10 (41.6%)	16 (43.3%)
Human strains	n = 7	n = 10	n = 17
*act-aer*A	1 (14.3%)	0	1 (5.9%)
*act-aer*A*-hly*A	0	1 (10%)	1 (5.9%)
*act-alt-aer*A	0	1 (10%)	1 (5.9%)
*aer*A	1 (14.3%)	0	1 (5.9%)
*aer*A*-hly*A	1 (14.3%)	3 (30%)	4 (23.5%)
*alt*-*aer*A	0	1 (10%)	1 (5.9%)
*alt*-*hly*A	1 (14.3%)	2 (20%)	3 (17.6%)
*hly*A	2 (28.5%)	1 (10%)	3 (17.6%)
Negative	1 (14.3%)	1 (10%)	2 (11.8%)
Animal strains	n = 14	n = 67	n = 81
*act*	1 (7.1%)	2 (3%)	3 (3.7%)
*act-aer*A	2 (14.3%)	4 (5.9%)	6 (7.4%)
*act-alt*	0	2 (3%)	2 (2.5%)
*act-alt*-*aer*A	1 (7.1%)	1 (1.5%)	2 (2.5%)
*act-alt*-*aer*A*-hly*A	4 (28.8%)	11 (16.4%)	15 (18.5%)
*act-alt*-*hly*A	1 (7.1%)	4 (5.9%)	5 (6.1%)
*act-hly*A	0	2 (3%)	2 (2.5%)
*aer*A	0	4 (5.9%)	4 (5%)
*aer*A*-hly*A	2 (14.3%)	6 (9%)	8 (9.9%)
*alt*	0	10 (15%)	10 (12.3%)
*alt-aer*A	0	1 (1.5%)	1 (1.2%)
*alt-aer*A*-hly*A	1 (7.1%)	3 (4.5%)	4 (5%)
*alt-hly*A	0	2 (3%)	2 (2.5%)
*hly*A	1 (7.1%)	2 (3%)	3 (3.7%)
Negative	1 (7.1%)	13 (19.4%)	14 (17.3%)

*act*—cytotoxic enterotoxin, *alt*—heat-labile cytotonic enterotoxin; *aer*A—aerolysin; *hly*A—hemolysin.

**Table 4:** Distribution of Antimicrobial Resistance in *A. dhakensis* and *A. hydrophila*.

Antimicrobial Drugs	No. (%) of Resistant Isolates		
	*Aeromonas dhakensis * N. 34	*Aeromonas hydrophila* N. 101	Total N. 135
Ceftazidime	4 (11.8%)	11 (10.9%)	15 (11.1%)
Cefotaxime	0	2 (1.98%)	2 (1.5%)
Cefoxitin	12 (35.3%)	29 (28.7%)	41 (30.4%)
Imipenem/Meropenem	10 (29.4%)	32 (31.7%)	42 (31.1%)
Gentamycin	3 (8.8%)	4 (3.96%)	7 (5.2%)
Ciprofloxacin	0	2 (1.98%)	2 (1.5%)
Nalidixic Acid	22 (64.2%)	48 (47.5%)	70 (69.3%)
Chloramphenicol	2 (5.9%)	11 (10.9%)	13 (9.6%)
Tetracycline	4 (11.8%)	16 (15.8%)	20 (14.8%)
Sulfamethoxazole-trimethoprim	6 (17.6%)	15 (14.8%)	21 (15.5%)
Nitrofurantoin	4 (11.8%)	12 (11.8%)	16 (11.8%)

N—number.

**Table 5:** Distribution of *Aeromonas* spp. MDR strains by source, virulence, and resistance profile.

Strain	Species	Source	Virulence Profile ^a^	MDR Profile ^b^
AdE01	*A. dhakensis*	Sewage water	*act*	CAZ-IPM-NAL-SXT
AdE02	*A. dhakensis*	Sewage water	act-aerA	IPM-NAL-SXT
AdE03	*A. dhakensis*	Sewage water	act-aerA-hlyA	IPM-NAL-TCY-NIT
AdE07	*A. dhakensis*	Sewage water	None	CAZ-IPM-NAL-SXT-NIT
AdA01	*A. dhakensis*	*Mugil liza*	act-alt-aerA-hlyA	NAL-CHL-TCY
AdA02	*A. dhakensis*	*Trichechus inunguis*	alt-aerA-hlyA	FOX-GEN-NIT
AdH01	*A. dhakensis*	Fecal swab	None	FOX-NAL-TCY
AhE22	*A. hydrophila*	Sewage water	act-hlyA	NAL-SXT-NIT
AhE23	*A. hydrophila*	Sewage water	aerA	NAL-TCY-SXT-NIT
AhE24	*A. hydrophila*	Sewage water	alt	IPM-NAL-TCY-SXT
AhA09	*A. hydrophila*	*Balaenoptera* sp.	None	CAZ-FOX-IMP-NIT
AhA12	*A. hydrophila*	*Isopisthus parvipinnis*	alt-aerA	CAZ-IPM-GEN-NAL-SXT
AhA13	*A. hydrophila*	*Macropogonias furnieri*	None	FOX-CHL-TCY
AhA19	*A. hydrophila*	*Mugil liza*	alt	IPM-NAL-TCY-SXT
AhA20	*A. hydrophila*	*Mugil liza*	alt	IPM-NAL-TCY-SXT
AhA30	*A. hydrophila*	*Mugil liza*	act-aerA	FOX-MEM-GEN
AhA40	*A. hydrophila*	*Mugil liza*	act-alt	FOX-IPM-NIT
AhA45	*A. hydrophila*	*Mugil liza*	act-alt-hlyA	FOX-NAL-SXT
AhA47	*A. hydrophila*	*Mugil liza*	None	FOX-IPM-NAL-NIT
AhA48	*A. hydrophila*	*Phalacrocorax brasilianus*	act	CAZ-NAL-CHL
AhA52	*A. hydrophila*	*Phalacrocorax brasilianus*	aerA	CAZ-CTX-CHL-TCY
AhA53	*A. hydrophila*	*Phalacrocorax brasilianus*	aerA	CAZ-FOX-NAL-NIT
AhA66	*A. hydrophila*	*Phalacrocorax brasilianus*	None	CAZ-FOX-CIP-NAL-CHL
AhA67	*A. hydrophila*	*Phalacrocorax brasilianus*	None	CHL-TCY-FOX

^a^ *act*—cytotoxic enterotoxin, *alt*—heat-labile cytotonic enterotoxin *aer*A—aerolysin gene, *hly*A—hemolysin gene. ^b^ Nalidixic Acid—NAL; Ceftazidime—CAZ; Cefoxitin—FOX; Ceftriaxone—CTX; Ciprofloxacin—CIP; Chloramphenicol—CHL; Gentamicin—GEN; Imipenem—IPM; Meropenem—MEM; Nitrofurantoin—NIT; Sulfamethoxazole-trimethoprim—SXT; and Tetracycline—TCY.

## Data Availability

The raw data supporting the conclusions of this article will be made available by the authors on request.
